# Effect of Thymoquinone on Acute Kidney Injury Induced by Sepsis in BALB/c Mice

**DOI:** 10.1155/2020/1594726

**Published:** 2020-06-16

**Authors:** Li-Peng Guo, Si-Xu Liu, Qin Yang, Hong-Yang Liu, Lu-Lu Xu, Yu-Hua Hao, Xiao-Qing Zhang

**Affiliations:** ^1^Department of Cardiology, Dalian Third People's Hospital Affiliated to Dalian Medical University, No. 40 Qianshan Road, Dalian, China; ^2^Department of Internal Medicine, Affiliated Zhongshan Hospital of Dalian University, No. 6 Jiefang Street, Dalian, China; ^3^Department of Heart Intensive Care Unit, The First Affiliated Hospital of Dalian Medical University, No. 193 Lianhe Road, Dalian, China; ^4^Department of Infection, Affiliated Zhongshan Hospital of Dalian University, No. 6 Jiefang Street, Dalian, China

## Abstract

Acute kidney injury (AKI) is a common complication of sepsis and has also been observed in some patients suffering from the new coronavirus pneumonia COVID-19, which is currently a major global concern. Thymoquinone (TQ) is one of the most active ingredients in *Nigella sativa* seeds. It has a variety of beneficial properties including anti-inflammatory and antioxidative activities. Here, we investigated the possible protective effects of TQ against kidney damage in septic BALB/c mice. Eight-week-old male BALB/c mice were divided into four groups: control, TQ, cecal ligation and puncture (CLP), and TQ+CLP. CLP was performed after 2 weeks of TQ gavage. After 48 h, we measured the histopathological alterations in the kidney tissue and the serum levels of creatinine (CRE) and blood urea nitrogen (BUN). We also evaluated pyroptosis (NLRP3, caspase-1), apoptosis (caspase-3, caspase-8), proinflammatory (TNF-*α*, IL-1*β*, and IL-6)-related protein and gene expression levels. Our results demonstrated that TQ inhibited CLP-induced increased serum CRE and BUN levels. It also significantly inhibited the high levels of NLRP3, caspase-1, caspase-3, caspase-8, TNF-*α*, IL-1*β*, and IL-6 induced by CLP. Furthermore, NF-*κ*B protein level was significantly decreased in the TQ+CLP group than in the CLP group. Together, our results indicate that TQ may be a potential therapeutic agent for sepsis-induced AKI.

## 1. Introduction

There was a rapid increase in the number of COVID-19 cases worldwide; this pandemic caused by a new human coronavirus is of global concern [1, 2]. Some patients progressed rapidly with septic shock, which was eventually followed by multiple organ failure [3]. Complications in patients besides acute respiratory distress syndrome and acute heart damage include acute kidney damage. AKI is a serious complication of sepsis associated with high morbidity and mortality [4, 5]. Increasing evidence suggests that oxidative stress, hypoxia, intrarenal inflammatory response, and renal cell apoptosis are the main mechanisms of sepsis-related renal injury [6]. Thymoquinone (TQ) is a pharmacologically active plant quinone found in black cumin seeds [7]. Antioxidant, anti-inflammatory, and immunomodulatory effects of TQ against renal damage have been demonstrated [8, 9]. Selçuk et al. demonstrated that TQ can promote burn wound healing by reducing inflammation and oxidative stress [10]. Farooqui et al. reported that oral administration of TQ effectively mitigated the renal damage caused by cisplatin-generated free radical attack [11]. Another study indicated that TQ could ameliorate renal toxicity induced by a high-dose diclofenac treatment [12]. However, the role of TQ in sepsis-induced AKI is not known. In the current study, we established the model of sepsis-induced AKI in BALB/c mice and investigated the effect and mechanism(s) of TQ on sepsis-induced AKI.

## 2. Material and Methods

### 2.1. Animals

All animal experiments were performed in accordance with the Guide for the Care and Use of Laboratory Animals. The study was approved by the ethical committee of the affiliated Zhongshan Hospital of Dalian University. Male BALB/c mice were obtained from Beijing Vital River Lab Animal Technology Co., Ltd. (Beijing, China). All mice were housed in a room at 23–25°C and 40–60% humidity, with a 12 h light/dark cycle.

### 2.2. Murine Model of Sepsis

All animals, except those in the sham group, were established as a classic sepsis-induced AKI model by the method of cecal ligation and puncture (CLP) [13]. Briefly, after anesthetizing with sodium pentobarbital (100 mg/kg intraperitoneal) and skin sterilization, a midline abdominal incision of approximately 2–3 cm was made to expose the cecum. Two-thirds of the cecum was tied off, then punctured once with a 21-gauge needle, and a small amount of cecal content was squeezed out through the puncture wound. Finally, the cecum was returned to the peritoneal cavity and the laparotomy site was stitched layer by layer. Comparatively, sham-operated mice were exposed and massaged as described above, but they were not ligated or punctured.

Forty-eight 8-week-old male mice were randomly divided into four groups (*n* = 12 each): control (sham-operated); TQ (50 mg/kg/day; Sigma-Aldrich, St. Louis, MO, USA), CLP, and TQ+CLP. CLP was performed after the mice were subjected to 2-week TQ gavage. All surviving animals were killed after forty-eight hours; blood samples were obtained from the inferior vena cava and collected in serum tubes, then stored at −80°C until further analysis. Longitudinal sections of the kidneys were fixed in 10% formalin and embedded in paraffin for histological evaluation. The remaining the kidney tissues were snap frozen in liquid nitrogen for mRNA or immunoblotting analysis.

### 2.3. Biochemical Measurements

Serum concentrations of creatinine (CRE) and blood urea nitrogen (BUN) were measured using an enzyme-linked immunosorbent assay kit (Westang, Shanghai, China).

### 2.4. Histological Analysis

Formalin-fixed and paraffin-embedded kidney tissues were cut into 4 *μ*m thick cross-sections. Serial sections were stained with hematoxylin and eosin (HE) and observed under an Olympus microscope (Olympus, Tokyo, Japan) to detect the lesion area in the kidney.

### 2.5. Morphological Analysis and Immunohistochemistry

The kidneys were isolated free from the surrounding connective tissue after sacrificing the mice. The kidney tissue was fixed with 4% paraformaldehyde, embedded in paraffin, and then cut into slices using a microtome (Leica RM 2235 or Leica CM1850UV; Leica, Solms, Germany). Immunohistochemistry was performed using a Histone Simple Stain Kit (Nichirei, Tokyo, Japan) according to the manufacturer's instructions. Briefly, serial sections (4 *μ*m thick) were deparaffinized with xylene, and then gradient ethanol was used to dewax and hydrate the samples. The sections were immersed in methanol with 3% H_2_O_2_ for 15 min to inactivate endogenous peroxidases and then incubated with primary antibodies at room temperature for 1 h. Primary antibodies against NLRP3 (rabbit anti-NLRP3 antibody, 1 : 200; Proteintech) and caspase-1 (rabbit anti-caspase-1 anti-body, 1 : 200; Proteintech) were used. All sections were visualized using an Olympus microscope (Olympus, Tokyo, Japan).

### 2.6. RNA Isolation and Real-Time RT-PCR

Total RNA was isolated from kidney tissues, and complementary DNA (cDNA) was prepared using the TransScript One-Step gDNA Removal and cDNA Synthesis SuperMix kit (TransGen, Beijing, China) according to the manufacturer's protocol. Quantitative reverse transcription–PCR was performed by TransStart Top Green qPCR SuperMix kit (TransGen). The relative amounts of the target genes were normalized by amplification and quantification *β*-actin cDNA in each cDNA preparation. The primers were as follows: IL-1*β*, forward primer 5′-TGCCACCTTTTGACAGTGAT-3′, reverse primer 5′-TGTGCTGCTGCGAGATTTGA-3′; IL-6, forward primer 5′-TACCAGTTGCCTTCTTGGGACTGA-3′, reverse primer 5′-TAAGCCTCCGACTTGTGAAGTGGT-3′; TNF-*α*, forward primer 5′-TCTCATGCACCACCATCAAGGACT-3′, reverse primer 5′-ACCACTCTCCCTTTGCAGAACTCA-3′; and *β*-actin, forward primer 5′-CGATGCCCTGAGGGTCTTT-3′, reverse primer 5′-TGGATGCCACAGGATTCCAT-3′.

### 2.7. Western Blotting Using Kidney Tissue

Proteins were extracted from kidney tissues using a radioimmunoprecipitation assay buffer (P0013B; Beyotime, Shanghai, China). An equivalent amount of each protein sample was loaded and separated through 10% SDS-PAGE gel and transferred onto polyvinylidene fluoride membrane (Immobilon, Millipore, Billerica, MA, USA). The membranes were blocked in a Tris-buffered saline with 0.1% Tween-20 (TBST) containing 5% skim milk, and then incubated the membranes with the primary antibody that was diluted using the primary antibody diluent (P0023A; Beyotime) overnight at 4°C. The primary antibodies used were against caspase-1 (rabbit anti-caspase-1 antibody, 1 : 500; Proteintech), caspase-3, caspase-8, NLRP3, NF-*κ*B (rabbit anti-caspase-3, anti-caspase-8, anti-NLRP3, and anti-NF-*κ*B antibody, 1 : 1000; Proteintech), and anti-*β*-actin (1 : 1000; Proteintech). Thereafter, the membranes were washed and then incubated with a secondary antibody (anti-rabbit Ig-G, 1 : 1000; Cell Signaling Technology) for 1 h at room temperature. Bound antibodies were detected with enhanced chemiluminescence reagent and the immunoreactive bands were imaged using a Bio-Rad imaging system with Chemi HR camera 410 and Gel-Pro Analyzer version 4.0 (Media Cybernetics, Rockville, MD, USA). Protein levels are expressed as protein/*β*-actin ratios to minimize loading differences. The protein bands were quantified by NIH ImageJ software. The analysis was carried out independently three times.

### 2.8. Statistical Analysis

All data are presented as the mean ± standard error of mean. The statistical analysis was made by using SPSS software version 23.0 (SPSS Inc., Chicago, IL, USA). Group differences were analyzed by one-way ANOVA and subsequent Tukey's test. *P* < 0.05 value was considered statistically significant.

## 3. Results

### 3.1. Metabolic Characterization

The metabolic characteristics of mice in the four different groups are shown in Table 1. Body/kidney body weights did not differ among the four groups. At 48 h after CLP injury, we observed a significant increase in serum BUN and CRE levels in the CLP group than in the control group, but treatment of TQ significantly decreased serum BUN and CRE levels.

### 3.2. TQ Reduced Inflammatory Reactions in the Kidney of CLP Group Mice

**Figure 1:**
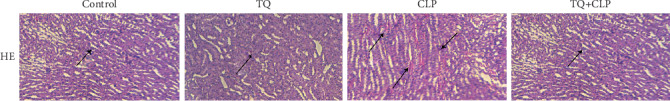
Inflammatory cell infiltration in kidney tissues from the BALB/c mice of the four groups with different treatments. The arrows indicated damage. Magnification 40x.

To evaluate tissue damage and inflammatory cell infiltration in the kidney tissues, HE staining was performed (Figure 1). The CLP group showed the infiltration of inflammatory cells into the kidney tissue, which could be ameliorated by treatment with TQ (as observed in the TQ+CLP group). This indicated that TQ could suppress inflammatory cell infiltration into the kidney tissue in the CLP-induced AKI model.

### 3.3. TQ Reduced Proinflammatory Cytokine Expression in Kidney Tissue of CLP Group Mice

**Figure 2:**
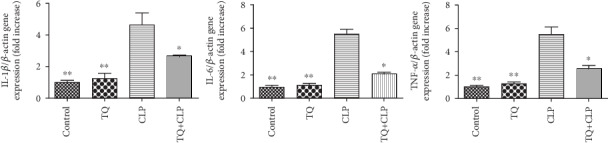
Relative mRNA expression levels of IL-1*β*, IL-6, and TNF-*α* expression in the kidney tissue of the four groups after different treatments. Data are means ± SEM; *n* = 6 per group. ^∗^*P* < 0.05 vs. the CLP group; ^∗∗^*P* < 0.01 vs. the CLP group.

To evaluate the involvement of proinflammatory cytokines in the kidney tissue of the four groups, interleukin- (IL-) 1*β*, IL-6, and tumor necrosis factor alpha (TNF-*α*) gene expression was measured by real-time PCR (Figure 2). All three genes were increased in the CLP group mice compared with the control group. However, this increase was attenuated in the TQ+CLP group.

### 3.4. TQ Reduced Pyroptosis-Related Expression in Kidney Tissue of CLP Group Mice

**Figure 3:**
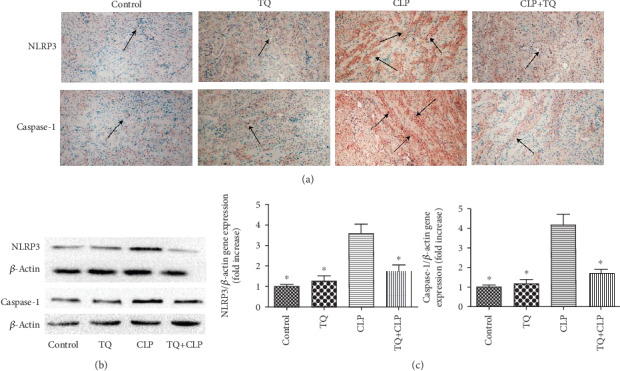
(a) Representative immunohistochemistry staining for NLRP3 and caspase-1 in kidney tissue of mice with different treatments. Magnification 40x. Arrows indicate positively stained cells (*n* = 3). (b) Immunoblotting for NLRP3 and caspase-1 in kidney tissue. (c) Bar graph showing quantification of NLRP and caspase-1protein expression. Data are means ± SEM; *n* = 3 per group. ^∗^*P* < 0.05 vs. the CLP group.

To investigate the mechanism of pyroptosis in kidney tissue, immunohistochemical assay was performed to analyze the expression of pyroptosis-related NLRP3 and caspase-1 proteins (Figure 3(a)). The TQ+CLP group showed greatly reduced NLRP3 and caspase-1 expression in the kidney tissue compared to the CLP group. Immunoblotting assay also showed a similar result, wherein the CLP-induced increase in the expression of these proteins was attenuated by TQ treatment (Figures 3(b) and 3(c)). Thus, TQ can reduce pyroptosis-related protein expression in the kidney tissue of the AKI model.

### 3.5. TQ Reduced Apoptosis-Related Expression in Kidney Tissue of CLP Group Mice

**Figure 4:**
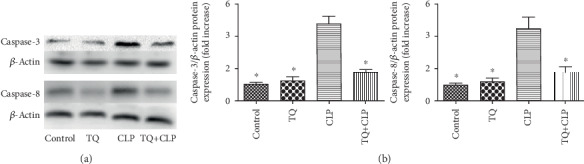
(a) Immunoblotting for caspase-3 and caspase-8 in kidney tissue. (b) Bar graph showing quantification of caspase-3 and caspase-8 protein expression. Data are means ± SEM; *n* = 3 per group. ^∗^*P* < 0.05 vs. the CLP group.

We next used immunoblotting to evaluate the expression of caspase-3 and caspase-8 proteins (Figure 4) and found that their expression levels were increased in the CLP group mice compared with those in the control group mice. Interestingly, this increase was suppressed with the treatment of TQ.

### 3.6. TQ Reduced Nuclear Transcription Factor-*κ*B (NF-*κ*B) in Kidney Tissue of CLP Group Mice

**Figure 5:**
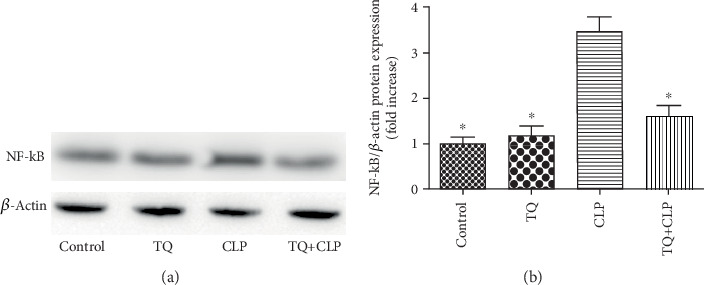
NF-*κ*B protein expression in the kidney tissue of the four groups after different treatments. (a) Immunoblotting analysis to detect the NF-*κ*B expression in the kidney tissues. (b) Bar graph depicting the semiquantification of the NF-*κ*B expression. TQ suppressed the expression of the NF-*κ*B protein in the CLP group mice. Data are means ± SEM; *n* = 3 per group. ^∗^*P* < 0.05 vs. the CLP group.

To investigate the effect of TQ on the regulation of the NF-*κ*B signaling pathway, immunoblotting with NF-*κ*B was performed (Figure 5). We found a higher expression of NF-*κ*B in the CLP group compared with that in the control group; however, this increase was markedly suppressed in the TQ+CLP group.

## 4. Discussion

This study demonstrates that TQ has a protective effect against AKI via the anti-inflammatory and antiapoptosis pathways. In the present study, the sepsis-induced AKI model was established by CLP surgery in BALB/c mice to investigate the renoprotective effects of TQ. With respect to metabolic characteristics, serum CRE and BUN levels are classical indices to evaluate kidney function. In our study, the CLP group showed significantly higher kidney indexes of kidney injury (CRE, BUN) compared to the control group. However, the addition of TQ significantly reduced the serum CRE and BUN levels, alleviating the sepsis-induced AKI. These results are in agreement with the report by Zhang et al. [14].

Sepsis is a systemic inflammatory response syndrome that can lead to multiple organ dysfunction. AKI is a common complication of sepsis [2], and inflammatory response is reported to play an important role in sepsis-related AKI [15, 16]. In our study, HE staining showed a higher inflammatory cell infiltration in the CLP group mice than in the control group mice. Interestingly, TQ reduced such infiltration in the TQ+CLP group. In addition, TQ acted by reducing sepsis-induced increase in the expression of inflammatory signals including that of IL-1-*β*, IL-6, and TNF-*α* in the kidney tissue. Kapan et al. show that TQ reduced the levels of IL-1-*β*, IL-6, and TNF-*α* in mice with intestinal obstruction, which is consistent with our results [17]. When the balance between the proinflammatory and anti-inflammatory responses during sepsis is broken, it permits excessive release of proinflammatory cytokines, thus inducing kidney injury [18]. Proinflammatory cytokines are major mediators of sepsis-induced AKI [19]. Therefore, inhibition of the release of proinflammatory cytokines may be an effective approach to treat sepsis-induced AKI [20]. Our studies demonstrated that the inhibition of proinflammatory factors could reduce sepsis-induced AKI.

NLRP3 is an inflammasome complex whose dysregulated activation is associated with AKI and sepsis [21]. NLRP3 was reported to regulate the maturation of IL-18 and IL-1*β*, via recruitment and activation of caspase-1, and thereby induce inflammatory cell death (pyroptosis) [22, 23]. Thus, NLRP3 could be a promising molecular target for sepsis-induced AKI treatment. To investigate the effect and mechanisms of TQ, we performed immunohistochemical and immunoblotting assays to analyze the expression of caspase-1 and NLRP3. Our results revealed that the TQ+CLP group had markedly reduced caspase-1 and NLRP3 expression in the kidney tissues compared to that in the CLP group. These results indicate that TQ downregulated caspase-1 and NLRP3 in the CLP group mice. Dai et al. established a classic septic model in rats by CLP and attenuated sepsis-induced renal injury by inhibiting NLRP3 and caspase-1 [24]; Liu et al. also found that TQ attenuated sepsis-induced cardiac injury by reducing the expression of NLRP3 and caspase-1 [25]; these results are in agreement with our research.

Apoptosis is one of the main mechanisms of sepsis-induced AKI [6]. Many studies have shown that inhibiting apoptosis (caspase-3, caspase-8) attenuates kidney injury [15, 26, 27]. In addition, a study by Lee et al. [28] found that caspase-3 inhibitors show some protective effect against the development of sepsis in experimental septic mouse model. We obtained similar results in our study by analyzing caspase-3 and caspase-8 expression in the kidney tissue with immunoblotting. The expression of these proteins was significantly increased in the CLP group compared with that in the control group, and it was markedly decreased in the TQ+CLP group compared with that in the CLP group. Also, this result is similar to the discovery of Hou and colleagues; they found that TQ decreased the levels of caspase-3 and caspase-8 and thereby decreases the apoptosis of the small intestine to reduce the severity of radiation-induced enteritis [29].

NF-*κ*B, as the main transcriptional regulator of inflammation-related genes, plays a significant role in inflammation [30]. Current studies on animal models of septic shock have confirmed that NF-*κ*B is important in the pathophysiology of sepsis [31–34]. In addition, the inhibition of NF-*κ*B was shown to reduce sepsis-induced kidney injury in mouse [35, 36]. Therefore, the present study evaluated the effect of TQ on NF-*κ*B and demonstrated that the expression of NF-*κ*B was markedly increased in the CLP group compared to that in the control group. Interestingly, additional TQ treatment reversed the increase in NF-*κ*B expression in the kidney from the septic mice.

## 5. Conclusions

Our study established that TQ contributes to the mitigation of sepsis-induced AKI by regulating proinflammatory cytokine and pyroptosis- and apoptosis-related expression. These findings provide a new strategy to treat AKI, including COVID-19-induced AKI.

## Figures and Tables

**Table 1:** Metabolic characteristic results.

	Control	TQ	CLP	TQ+CLP
Body weight (g)	24.12 ± 1.63	25.33 ± 1.06	23.23 ± 0.81	24.63 ± 1.21
Kidney/body weight (mg/g)	6.82 ± 1.35	6.23 ± 0.57	6.11 ± 0.22	6.13 ± 0.69
BUN (mg/dl)	22.37 ± 1.96^∗∗^	25.42 ± 2.43^∗∗^	107.62 ± 5.61	66.19 ± 4.36^∗^
CRE (mmol/ml)	49.24 ± 7.83^∗^	53.21 ± 6.91^∗^	156.87 ± 11.57	63.28 ± 7.87^∗^

Data are means ± SEM; *n* = 8 per group. ^∗^*P* < 0.05 vs. the CLP group; ^∗∗^*P* < 0.01 vs. the CLP group.

## Data Availability

All datasets are available from the corresponding author upon reasonable request.
