# Erratum to “Effect of Thymoquinone on Acute Kidney Injury Induced by Sepsis in BALB/c Mice”

**DOI:** 10.1155/2020/3182919

**Published:** 2020-11-24

**Authors:** Li-Peng Guo, Si-Xu Liu, Qin Yang, Hong-Yang Liu, Lu-Lu Xu, Yu-Hua Hao, Xiao-Qing Zhang

**Affiliations:** ^1^Department of Cardiology, Dalian Third People's Hospital Affiliated to Dalian Medical University, No. 40 Qianshan Road, Dalian, China; ^2^Department of Internal Medicine, Affiliated Zhongshan Hospital of Dalian University, No. 6 Jiefang Street, Dalian, China; ^3^Department of Heart Intensive Care Unit, The First Affiliated Hospital of Dalian Medical University, No. 193 Lianhe Road, Dalian, China; ^4^Department of Infection, Affiliated Zhongshan Hospital of Dalian University, No. 6 Jiefang Street, Dalian, China

In the article titled “Effect of Thymoquinone on Acute Kidney Injury Induced by Sepsis in BALB/c Mice” [1], Figure 1 contains an error where the image of the control group was duplicated in the image for the TQ+CLP group. This duplication was introduced during the production of the article, and the publisher apologises for this error. The corrected figure is shown below and is listed as Figure 1:

## Figures and Tables

**Figure 1:**
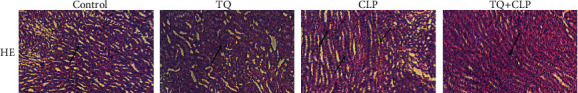
Inflammatory cell infiltration in kidney tissues from the BALB/c mice of the four groups with different treatments. The arrows indicated damage. Magnification 40x.
